# Mechanistic insights into the multi-target anti-atherosclerotic actions of rhubarb: a traditional remedy revisited

**DOI:** 10.1186/s13020-025-01231-w

**Published:** 2025-10-16

**Authors:** Feng-jiao Hu, Yue-ming Tang, Bin-yue Hu, Feng-ning Yang, Wei Jin, Yi-fan Miao, Yun Lu

**Affiliations:** 1 Department of Emergency Medicine, Hospital of Chengdu University of Traditional Chinese Medicine, No. 39 Shi-Er-Qiao Road, Chengdu, 610072 Sichuan Province People’s Republic of China; 2 School of Clinical Medicine, Chengdu University of Traditional Chinese Medicine, Chengdu, China

**Keywords:** Rhubarb, Atherosclerosis, Chemical constituents, Pharmacological action

## Abstract

Atherosclerosis (AS), a chronic inflammatory vascular disease, underlies the pathogenesis of most cardiovascular disorders. Its pathologic features include endothelial damage, inflammatory response, lipid plaque formation and rupture, thrombosis, and arterial stenosis occlusion. Severe AS can lead to the development of stroke, myocardial infarction and other adverse outcomes. Therefore, it is essential to choose reliable and effective drugs to control or alleviate the deterioration of AS. Rhubarb is a kind of Chinese herb in the Polygonaceae family, with the scientific name of *Rheum palmatum* L. Numerous studies in recent years have confirmed that the Chinese herb rhubarb possesses a wide range of pharmacological activities against a variety of diseases, including inflammation, cancer, digestive disorders, and cardiovascular diseases. This paper focuses on the different pharmacological impacts and possible action mechanisms of the main active constituents of rhubarb (including anthraquinones, anthracenes, stilbenes, tannins, etc.) in the treatment of AS, providing useful references for the treatment of AS and innovative pharmaceutical development.

## Introduction

Atherosclerosis (AS) is a chronic inflammatory vascular disease characterized by lipid deposition and accumulation of inflammatory substances in the arterial wall, leading to progressive vascular damage [1, 2]. As the primary pathophysiological foundation of cardiovascular and cerebrovascular disorders, AS imposes a substantial global disease burden. According to the Global Burden of Disease Study 2021, approximately 20.5 million cardiovascular disease-related deaths occurred worldwide, with 85–90% attributable to atherosclerotic cardiovascular disease (ASCVD), making it the leading cause of cardiovascular mortality [3]. Notably, China has experienced a steadily increasing ASCVD burden in recent decades [3, 4]. Currently, ASCVD-related conditions (particularly stroke and ischemic heart disease) have become the predominant cause of death in both urban and rural Chinese populations, accounting for over 40% of total mortality [4]. Current clinical management primarily relies on statins and antiplatelet therapy, yet two major limitations persist.

First, approximately 25–40% of patients exhibit "residual risk", manifested as persistent low-grade inflammation (hs-CRP ≥ 2 mg/L) or plaque instability (as evidenced by subgroup analyses from the CANTOS study); second, prolonged medication use may result in adverse effects including new-onset diabetes and hepatic dysfunction [5, 6]. This therapeutic challenge has shifted research focus toward traditional medicines possessing multitarget regulatory potential [7]. Among numerous investigational candidates, rhubarb (*Rheum palmatum* L.) is a promising therapeutic drug with a variety of pharmacological effects. Documented in *Shennong’s Classic of Materia Medica* for its blood-activating and stasis-resolving properties, rhubarb contains various bioactive compounds with multi-system pharmacological activities [8, 9], demonstrating considerable potential in AS treatment [10, 11]. This review systematically examines the pharmacological effects and mechanistic basis of active components derived from rhubarb for AS management, offering new therapeutic perspectives and potential treatment options for this disease.

## The pathogenesis of atherosclerosis

AS represents a persistent inflammatory disorder with multifactorial etiology, which progresses through intertwined pathological mechanisms including dyslipidemia, endothelial dysfunction, oxidative stress, and dysregulated immune-inflammatory responses [12, 13]. The disease initiation is triggered by endothelial injury induced by hemodynamic changes (e.g., hypertension) or metabolic disturbances (e.g., hypercholesterolemia, hyperglycemia) [13]. Vascular smooth muscle cells (VSMCs) undergo phenotypic modulation from a contractile to synthetic state, migrating into the intima and proliferating, which contributes to lesion expansion by producing extracellular matrix proteins and cytokines. Subsequent subendothelial accumulation of low-density lipoprotein (LDL) particles promotes their oxidation (oxidized LDL, ox-LDL), a pivotal event that recruits circulating monocytes into the intima. These monocytes differentiate into macrophages, internalize ox-LDL primarily via scavenger receptors (e.g., CD36), and transform into lipid-laden foam cells—the pathological hallmark of early atherosclerotic lesions [14]. Persistent foam cell formation drives a self-sustaining inflammatory cascade, mediated by pro-inflammatory cytokines and smooth muscle cell proliferation, ultimately leading to fibrous cap formation. In advanced stages, plaques evolve into a necrotic core encapsulated by a fibrous layer, with VSMC-derived osteoblast-like cells secreting bone matrix proteins (e.g., alkaline phosphatase, osteopontin) that drive vascular calcification, a key indicator of plaque vulnerability. Calcification and intraplaque hemorrhage further destabilize plaques. Plaque rupture exposes thrombogenic substrates (e.g., tissue factor), precipitating thrombotic occlusion and acute cardiovascular events including ischemic stroke or myocardial infarction [15]. Collectively, AS progression exemplifies a dynamic crosstalk among metabolic dysregulation, chronic inflammation, and maladaptive vascular remodeling.

## Main chemical components and pharmacokinetic characteristics of rhubarb

### The main chemical components of rhubarb

**Fig. 1:**
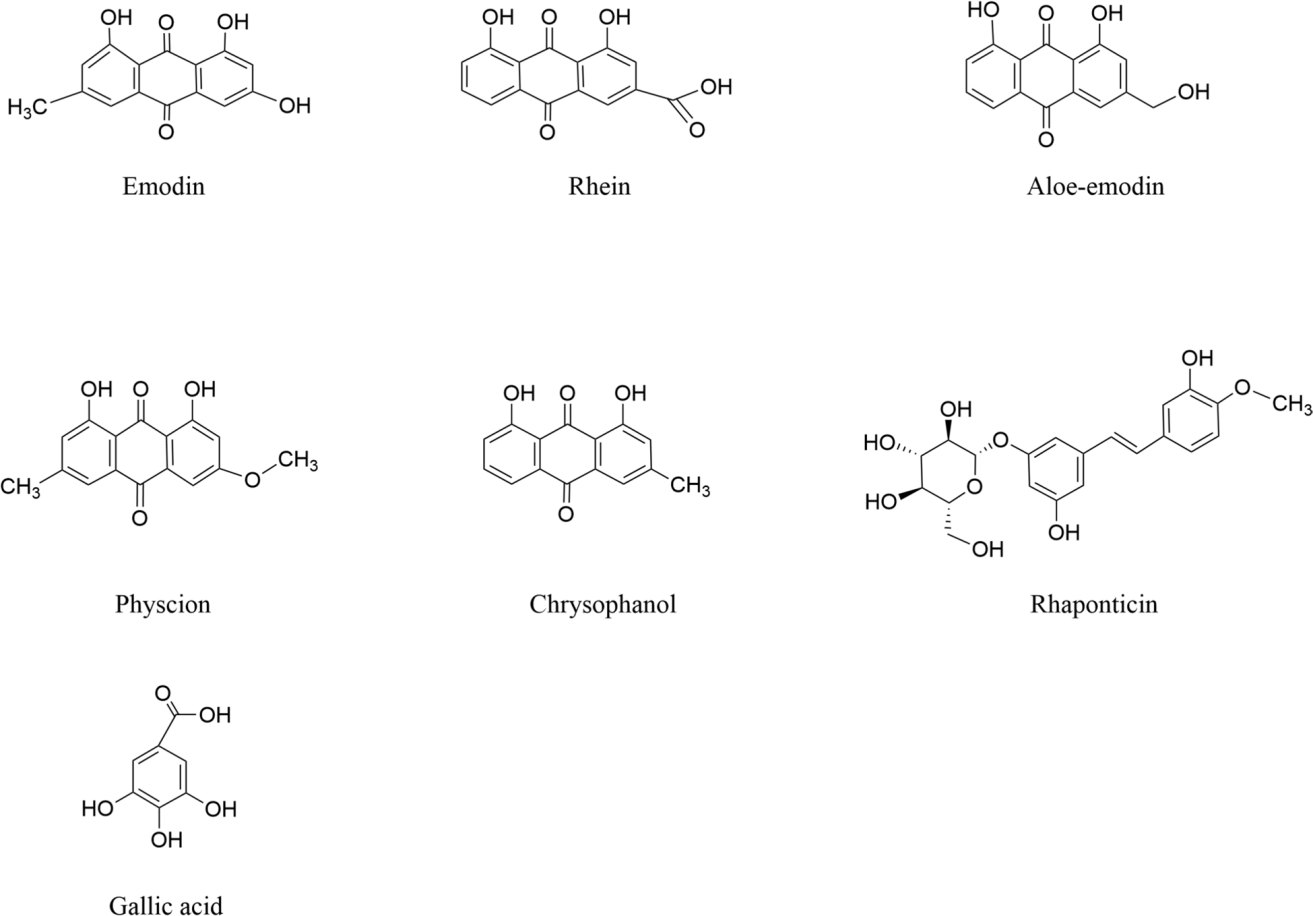
Chemical structure diagram of the main compounds of rhubarb

**Fig. 2:**
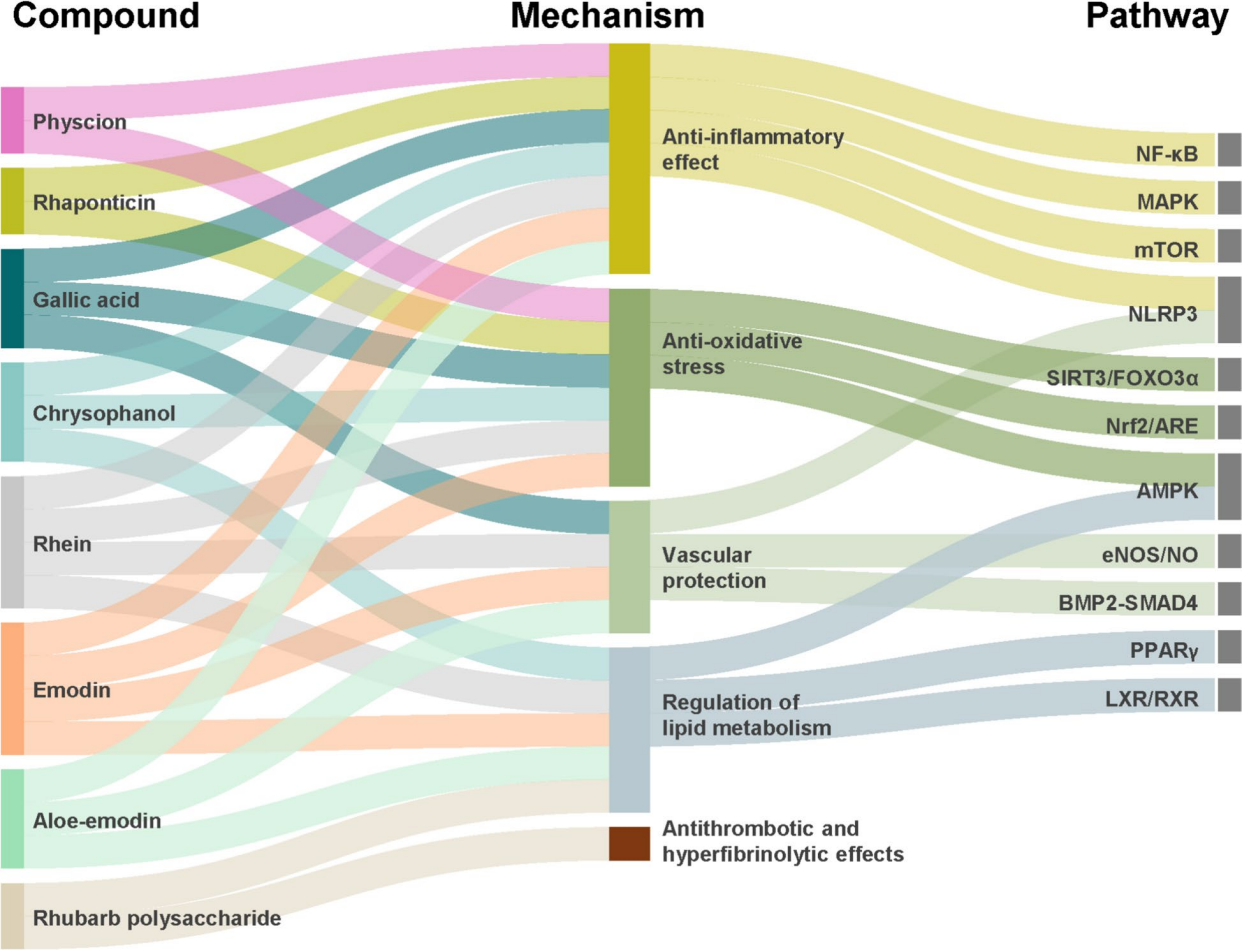
Mechanistic network diagram of active ingredients in rhubarb for the treatment of atherosclerosis. AMPK, Adenosine 5'-monophosphate (AMP)-activated protein kinase; ARE, antioxidant response element; BMP2, bone morphogenetic protein 2; eNOS, endothelial nitric oxide synthase; FOXO3α, forkhead box O3α; LXR, liver X receptor; MAPK, mitogen-activated protein kinase; mTOR, mammalian target of rapamycin; NF-κB, nuclear factor-kappa B; NLRP3, NOD-like receptor family pyrin domain containing 3; NO, nitric oxide; Nrf2, nuclear factor erythroid 2-related factor 2; PPARγ, peroxisome proliferator-activated receptor gamma; RXR, retinoid X receptor; SIRT3, sirtuin 3; SMAD4, mothers against decapentaplegic homolog 4

Given that the pathogenesis of AS is multifactorial, rhubarb, as a natural product capable of modulating multiple biological effects, has emerged as a promising candidate for intervention for AS (Fig. 1). Modern phytochemical studies have identified over 100 organic compounds in rhubarb [16], among which anthraquinones, tannins, and polysaccharides demonstrate the most significant biological activities (Table 1). Anthraquinone derivatives constitute the predominant phytochemicals, existing in both free forms (including emodin, rhein, aloe-emodin, and chrysophanol) and bound forms as glycosides or dimeric glycosides. As a subclass of anthraquinones, anthrones serve as the primary laxative components, with approximately 27 distinct derivatives identified to date that substantially contribute to the pharmacological effects of rhubarb [17]. The tannin fraction consists of condensed polyphenolic complexes formed through polymerization of gallic acid and catechins [18]. The polysaccharides are mainly composed of glucose, glucuronic acid, and galactose units [19]. Additional components include low-molecular-weight organic acids (such as palmitic acid and linoleic acid) and essential trace elements [20]. Through multi-target mechanisms involving lipid metabolism regulation and inflammatory pathway modulation, these compounds collectively exhibit therapeutic effects relevant to AS management (Fig. 2).

**Table 1:** Chemical composition of rhubarb and related information

Classification	Number	Name	Chemical formula	Molecular weight	References
Anthraquinone	1	Emodin	C_15_H_10_O_5_	270.24 g/mol	[162]
	2	Rhein	C_15_H_8_O_6_	284.22 g/mol	[163]
	3	Aloe-emodin	C_15_H_10_O_5_	270.24 g/mol	[164]
	4	Physcion	C_16_H_12_O_5_	284.26 g/mol	[165]
	5	Chrysophanol	C_15_H_10_O_4_	254.24 g/mol	[166]
	6	Rhaponticin	C_21_H_24_O_9_	420.40 g/mol	[167]
	7	Sennoside A	C_42_H_38_O_20_	862.70 g/mol	[168]
	8	Sennoside B	C_42_H_38_O_20_	862.70 g/mol	[169]
Tannins	9	Gallic acid	C_7_H_6_O_5_	170.12 g/mol	[170]
	10	Catechin	C_15_H_14_O_6_	290.27 g/mol	[171]
Polysaccharides	11	Rhubarb polysaccharide	(C_6_H_10_O_5_)_n_	variable	[172]

### The pharmacokinetic characteristics of rhubarb

The pharmacokinetic behaviors of anthraquinones, tannins, and polysaccharides in rhubarb exhibit marked differences, collectively forming the material basis for AS effects. Anthraquinones (e.g., emodin, aloe-emodin) are rapidly absorbed orally and widely distributed to the liver, kidneys, and vascular tissues [21]. Their metabolism primarily involves Phase II conjugation reactions (glucuronidation/sulfation), with conjugated metabolites excreted via urine and feces, often showing multi-peak phenomenon [22]. These compounds directly regulate lipid metabolism and inflammatory responses. Unlike anthraquinones, tannins and polysaccharides are not easily absorbed in the intestinal lumen. Tannins, owing to their high molecular weight and structural complexity, demonstrate minimal gastrointestinal absorption and low bioavailability, rarely entering systemic circulation [23]. They exert local anti-inflammatory effects in the gut, indirectly modulating lipid absorption [24]. Polysaccharides resist direct absorption, remaining in the intestinal lumen where gut microbiota ferment them into short-chain fatty acids and other metabolites. These metabolites enter systemic circulation, remodeling the gut-immune axis to achieve immunomodulation and endothelial protection [25]. To gain a deeper insight into the pharmacokinetic distinctions among anthraquinones in rhubarb, we present in Table 2 the key pharmacokinetic parameters for a selection of anthraquinone compounds. It is important to note that data for tannins and polysaccharides are currently incomplete. This is primarily due to the challenges associated with their detection, limitations in quantification methods, and the complexity of their metabolic pathways (Table 2). Notably, herbal compatibility optimizes pharmacokinetics by addressing limitations of single-drug use. In Sanhuang Xiexin Decoction, the combination of rhubarb with Coptis root (*Coptis chinensis Franch.*) and baical skullcap root (*Scutellaria baicalensis Georgi*) may enhance the immune memory of peripheral lymphocytes and natural killer cells, thereby achieving sustained anti-inflammatory effects [26, 27]. Compatibility with prepared aconite root (*Aconitum carmichaelii Debeaux*) at a 2:1 ratio significantly improves bioavailability of active components (e.g., aloe-emodin), while reducing cold-natured constituents (e.g., chrysophanol), balancing efficacy and safety. Prolonged exposure of key metabolites further synergizes to achieve "efficacy enhancement with toxicity reduction" [28]. Borneol (*Borneolum*) as a guiding agent enhances brain-targeted distribution and increases blood–brain barrier permeability, synergistically improving cerebral AS [29].

**Table 2:** Certain pharmacokinetic parameters of anthraquinones

Extract/compound	Treatment	Subject	Pharmacokinetic parameters	Reference
Aloe-emodin	13.05 mg/kg, i.g, qd × 7d	Male SD rats	t_1/2_ (h) = 14.98 ± 6.48, T_max_ (h) = 0.32 ± 0.12, C_max_ (ng/ml) = 9.88 ± 2.9, AUC_0-t_ (ng·h/ml) = 42.77 ± 10.09, AUC_0-∞_ (ng·h/ml) = 63.18 ± 24.20, AUMC_0-∞_ (ng·h^2^/ml) = 1524 ± 566.3, MRT_0-∞_ (h) = 21.73 ± 8.35, V/F (L) = 4287 ± 1948, Cl/F (L/h) = 244.2 ± 118.0	[173]
Emodin	34.65 mg/kg, i.g, qd × 7d	Male SD rats	t_1/2_ (h) = 23.13 ± 3.56, T_max_ (h) = 0.75 ± 0.00, C_max_ (ng/ml) = 91.65 ± 16.82, AUC_0-t_ (ng·h/ml) = 624.7 ± 73.35, AUC_0-∞_ (ng·h/ml) = 1108 ± 191.1, AUMC_0-∞_ (ng·h^2^/ml) = 33,673 ± 10,503, MRT_0-∞_ (h) = 29.90 ± 4.71, V/F (L) = 1052 ± 119.8, Cl/F (L/h) = 31.99 ± 5.15	
Rhein	17.25 mg/kg, i.g, qd × 7d	Male SD rats	t_1/2_ (h) = 10.20 ± 2.50, T_max_ (h) = 0.23 ± 0.06, C_max_ (ng/ml) = 19,290 ± 5420, AUC_0-t_ (ng·h/ml) = 18,866 ± 1739, AUC_0-∞_ (ng·h/ml) = 20,893 ± 2262, AUMC_0-∞_ (ng·h^2^/ml) = 157,175 ± 52,352, MRT_0-∞_ (h) = 7.44 ± 1.87, V/F (L) = 12.08 ± 2.07, Cl/F (L/h) = 0.83 ± 0.09	
Chrysophanol	39.45 mg/kg, i.g, qd × 7d	Male SD rats	t_1/2_ (h) = 37.47 ± 14.54, T_max_ (h) = 0.54 ± 0.10, C_max_ (ng/ml) = 19.01 ± 3.93, AUC_0-t_ (ng·h/ml) = 88.97 ± 10.58, AUC_0-∞_ (ng·h/ml) = 185.2 ± 56.36, AUMC_0-∞_ (ng·h^2^/ml) = 8755 ± 3200, MRT_0-∞_ (h) = 42.24 ± 23.55, V/F (L) = 10,372 ± 2880, Cl/F (L/h) = 236.8 ± 97.59	
Physcion	26.40 mg/kg, i.g, qd × 7d	Male SD rats	t_1/2_ (h) = 30.47 ± 14.4, T_max_ (h) = 0.38 ± 0.14, C_max_ (ng/ml) = 6.24 ± 0.59, AUC_0-t_ (ng·h/ml) = 36.38 ± 5.58, AUC_0-∞_ (ng·h/ml) = 72.50 ± 41.70, AUMC_0-∞_ (ng·h^2^/ml) = 4492 ± 2151, MRT_0-∞_ (h) = 41.50 ± 17.65, V/F (L) = 13,170 ± 6030, Cl/F (L/h) = 433.4 ± 161.6	
Chrysophanol	7.38 mg/kg, p.o, sd	Male SD rats	t_1/2_ (h) = 3.18 ± 0.62, T_max_ (h) = 2.22 ± 1.76, C_max_ (ng/ml) = 1.90 ± 0.56, AUC_0–t_ (ng·h/ml) = 4.28 ± 1.64, AUC_0–∞_ (ng·h/ml) = 10.3 ± 1.87, MRT_0–∞_ (h) = 6.76 ± 2.06	[174]
Emodin	14.8 mg/kg, p.o, sd	Male SD rats	t_1/2_ (h) = 8.37 ± 4.17, T_max_ (h) = 0.19 ± 0.09, C_max_ (ng/ml) = 175 ± 33.8, AUC_0–t_ (ng·h/ml) = 686 ± 187, AUC_0–∞_ (ng·h/ml) = 801 ± 233, MRT_0–∞_ (h) = 11.8 ± 5.27	
Aloe-emodin	13.7 mg/kg, p.o, sd	Male SD rats	t_1/2_ (h) = 3.44 ± 1.40, T_max_ (h) = 0.22 ± 0.07, C_max_ (ng/ml) = 11.3 ± 3.10, AUC_0–t_ (ng·h/ml) = 7.28 ± 3.25, AUC_0–∞_ (ng·h/ml) = 8.46 ± 3.44, MRT_0–∞_ (h) = 3.17 ± 1.54	
Rhein	8.82 mg/kg, p.o, sd	Male SD rats	t_1/2_ (h) = 1.18 ± 0.39, T_max_ (h) = 0.46 ± 0.10, C_max_ (ng/ml) = 1.06 ± 0.24, AUC_0–t_ (ng·h/ml) = 1.11 ± 0.63, AUC_0–∞_ (ng·h/ml) = 2.31 ± 0.51, MRT_0–∞_ (h) = 1.83 ± 0.56	
Emodin	33.40 mg/kg, i.g, sd	Male SD rats	C_max_ (mg/ml) = 6.05 ± 1.62, T_max_ (h) = 3.17 ± 0.75, t_1/2z_ (h) 7.16 ± 2.52, AUC_0–t_ (mg·h/ml) = 61.88 ± 14.64, MRT_0–t_ (h) = 8.63 ± 1.05, VRT_0–t_ (h^2^) = 32.89 ± 4.98	[175]
Rhein	34.50 mg/kg, i.g, sd	Male mice	C_max_ (μg/ml) = 2.311 ± 0.923, T_max_ (h) = 0.485 ± 0.154, t_1/2_ (h) 9.028 ± 3.782, AUC_0–t_ (μg·h/ml) = 8.043 ± 2.717, MRT_0–t_ (h) = 7.790 ± 3.051, CL (L/kg·h) = 3.596 ± 1.134, Vd (L/kg) = 46.853 ± 21.573	[176]
Chrysophanol	0.66 mg/g, p.o	Male SD rats	t_1/2_ (h) = 6.12 ± 72.37, T_max_ (h) = 0.333 ± 70.071, C_max_ (ng/ml) = 1189.25 ± 333.4, AUC_0-t_ (ng·h/ml) = 642.21 ± 119.51, AUC_0-∞_ (ng·h/ml) = 650.58 ± 126.26	[177]
Emodin	0.02 mg/g, p.o	Male SD rats	t_1/2_ (h) = 6.40 ± 3.31, T_max_ (h) = 0.333 ± 0.059, C_max_ (ng/ml) = 38.48 ± 3.15, AUC_0-t_ (ng·h/ml) = 39.75 ± 2.30, AUC_0-∞_ (ng·h/ml) = 42.83 ± 6.38	
Aloe-emodin	0.04 mg/g, p.o	Male SD rats	t_1/2_ (h) = 24.62 ± 7.27, T_max_ (h) = 0.333 ± 0.009, C_max_ (ng/ml) = 79.20 ± 34.76, AUC_0-t_ (ng·h/ml) = 80.84 ± 50.76, AUC_0-∞_ (ng·h/ml) = 106.79 ± 71.88	
Rhein	0.08 mg/g, p.o	Male SD rats	t_1/2_ (h) = 10.30 ± 5.02, T_max_ (h) = 0.333 ± 0.09, C_max_ (ng/ml) = 152.70 ± 23.91, AUC_0-t_ (ng·h/ml) = 199.81 ± 22.47, AUC_0-∞_ (ng·h/ml) = 208.63 ± 26.50	
Physcion	0.30 mg/g, p.o	Male SD rats	t_1/2_ (h) = 10.46 ± 1.46, T_max_ (h) = 0.167 ± 0.002, C_max_ (ng/ml) = 461.85 ± 266.77, AUC_0-t_ (ng·h/ml) = 218.42 ± 15.08, AUC_0-∞_ (ng·h/ml) = 224.41 ± 16.84	

Multiple studies have systematically investigated the multifaceted impact of disease states, animal models, and gender on the pharmacokinetics of rhubarb, while elucidating how these factors modulate pharmacodynamic processes to ultimately influence therapeutic outcomes. Notably, disease-specific alterations in drug metabolism pathways demonstrate significant variability: Hepatic impairment significantly disrupts anthraquinone glucuronidation, drastically reducing metabolic clearance and abolishing therapeutic effects. Conversely, diabetic nephropathy models demonstrate preserved pharmacokinetic profiles, with renoprotection maintained despite disease progression [30]. Interspecies differences are statistically significant (*P* < 0.05): aloe-emodin exhibits relative bioavailability in the order rat > mouse > dog. Rhein shows higher C_max_ and AUC in dogs compared to rats and mice. Both chrysophanol and physcion present higher C_max_ and AUC in mice and rats than in dogs, whereas no significant difference in emodin absorption is noted across the three animal species [31, 32]. Collectively, these findings highlight that pharmacokinetic behavior of rhubarb is not static but is dynamically shaped by biological contexts, including metabolic perturbations induced by disease and inherent species-specific characteristics. This observation emphasizes the importance of context-aware study design in preclinical research to enhance the translational relevance of therapeutic strategies based on rhubarb.

Additionally, sex-based differences in the absorption of chemical constituents from rhubarb are emerging as a pivotal focus in the pharmacokinetic research of traditional Chinese medicine (TCM). In a human trial involving 24 healthy volunteers (12 males and 12 females), following single-dose oral administration of a rhubarb aqueous extract, women exhibited significantly higher C_max_ for rhein (4.06 ± 1.23 μg/ml vs. 3.24 ± 1.06 μg/ml in men), along with prolonged t_1/2β_ (4.34 ± 1.02 h vs. 2.44 ± 1.85 h), extended MRT (7.91 ± 2.03 h vs. 4.27 ± 1.28 h), and elevated AUC_0-∞_ (2197.56 ± 278.91 μg/ml·min vs. 1754.82 ± 419.23 μg/ml·min), suggesting slower metabolic clearance in females [33]. Animal studies further revealed that female rats demonstrated significantly higher hepatic concentrations of rhein, emodin, and aloe-emodin compared to males, accompanied by prolonged drug accumulation and persistent residual signals post-administration [34, 35]. These disparities may be attributed to estrogen-mediated regulation of UDP-glucuronosyltransferase (a key enzyme involved in drug metabolism by facilitating the transfer of glucuronic acid to drugs, thereby increasing their water solubility for excretion) metabolic enzymes, altered volume of distribution due to higher adipose tissue proportions in females, and sex-specific differences in glomerular filtration rates. Given the increased susceptibility of women to drug accumulation and associated hepatotoxicity risks, clinical pharmacotherapy must rigorously account for sex-based pharmacokinetic variations to optimize therapeutic outcomes. These multidimensional pharmacokinetic characteristics provide a core foundation for elucidating the multi-target synergistic mechanisms of rhubarb formulations against AS and for optimizing therapeutic strategies.

## The effect of rhubarb on atherosclerosis

### Anti-inflammatory effect

**Fig. 3:**
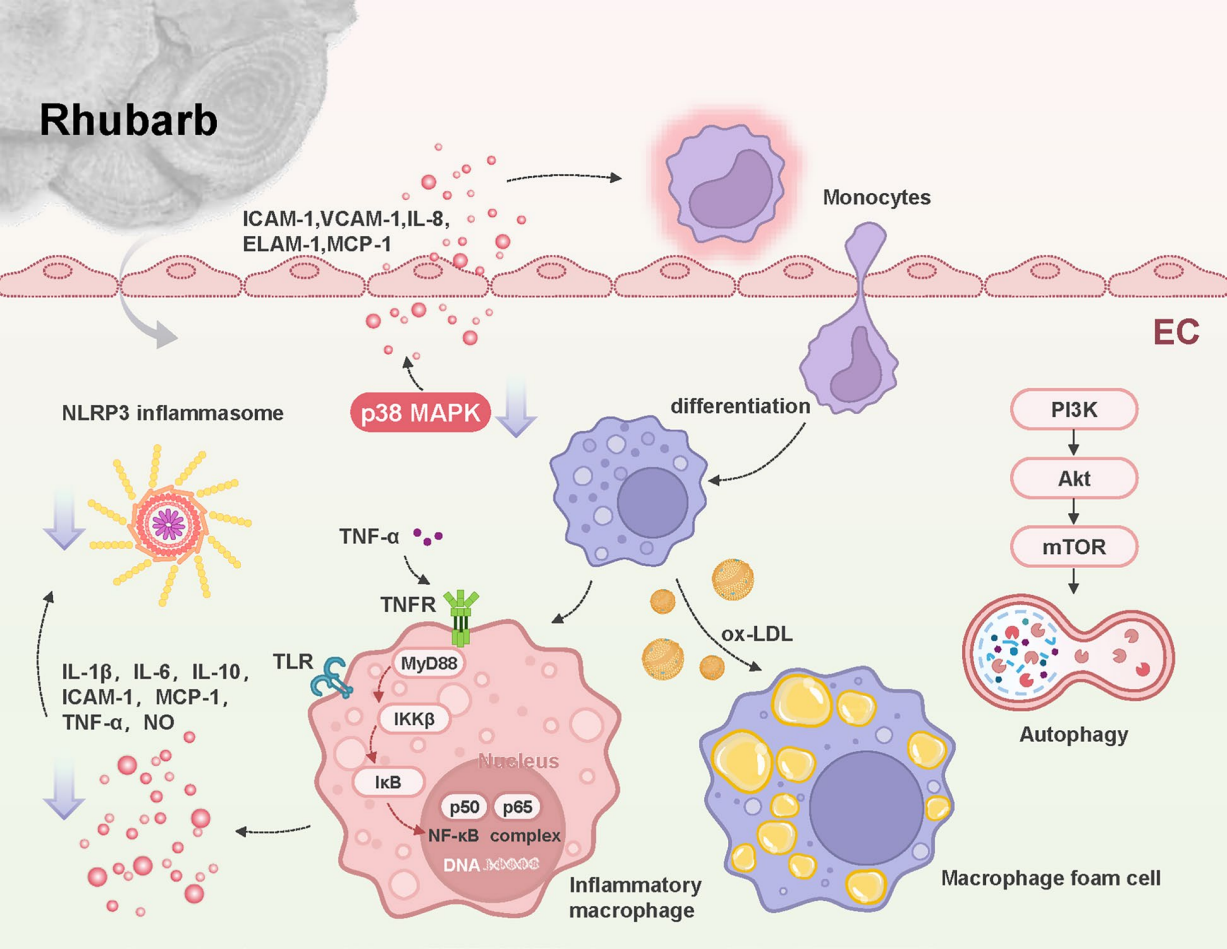
Anti-inflammatory pathways of rhubarb. Rhubarb and its chemical constituents exert robust anti-inflammatory effects via three key signaling pathways. Notably, they inhibit the NF-κB pathway to reduce pro-inflammatory factor production, directly attenuating inflammatory responses. Additionally, blocking the p38 MAPK pathway impedes immune cell transformation into foam cells, a process that supports atherosclerosis prevention. Furthermore, suppressing the PI3K/Akt/mTOR pathway induces autophagy; this process indirectly ameliorates the inflammatory environment, further enhancing overall anti-inflammatory and disease-modifying effects. Akt, protein kinase B; EC, endothelial cell; ELAM-1, endothelial-leukocyte adhesion molecule 1; ICAM-1, intercellular adhesion molecule-1; IKKβ, IκB kinase β; IκB, Inhibitor of κB; IL-1β, interleukin-1β; IL-6, interleukin-6; IL-8, interleukin-8; IL-10, interleukin-10; MAPK, mitogen-activated protein kinase; MCP-1, monocyte chemoattractant protein-1; mTOR, mammalian target of rapamycin; MyD88, myeloid differentiation primary response protein 88; NLRP3, NOD-like receptor family pyrin domain containing 3; NO, nitric oxide; ox-LDL, oxidized low-density lipoprotein; PI3K, phosphatidylinositol 3-kinase; TLR, Toll-like receptor; TNF-α, tumor necrosis factor-alpha; TNFR, Tumor necrosis factor receptor;VCAM-1, vascular cell adhesion molecule-1

The pathogenesis of AS is characterized by a disrupted equilibrium between pro-inflammatory and anti-inflammatory factors, mediated through complex interactions among immune cells and vascular components [36,37]. Pharmacological studies have documented the remarkable anti-inflammatory properties of rhubarb in AS models [38]. Specifically, in apolipoprotein E-deficient (*ApoE-/-*) mice (a widely used model for spontaneous AS due to disrupted lipoprotein metabolism), wine-processed rhubarb extract markedly reduced the production of tumor necrosis factor-α (TNF-α) and monocyte chemoattractant protein-1 (MCP-1) in atherosclerotic lesions, and concurrently reduced plaque formation while enhancing plaque stability, indicating that rhubarb has therapeutic potential for AS by modulating inflammatory pathways [39]. The subsequent section examines the detailed molecular mechanisms through which the key active compounds in rhubarb mediate their anti-inflammatory actions (Fig. 3).

#### Rhein

Over the past decade, extensive in vivo and in vitro studies have demonstrated the potent anti-inflammatory properties of rhein. Notably, rhein reduces the levels of pro-inflammatory mediators such as interleukin-1β, lipopolysaccharide (LPS), and TNF-α. It also inhibits the production of key proteins involved in critical signaling pathways. For instance, rhein targets the phosphatidylinositol 3-kinase (PI3K) gamma pathway, which is essential for cell growth and survival, and the Janus kinase 2 pathway, a key regulator of immune responses. In addition, rhein suppresses the production of matrix metalloproteinases (enzymes that degrade the extracellular matrix during inflammation and contribute to tissue remodeling). By interfering with these signaling processes, rhein blocks inflammatory cascades and modulates the expression of downstream proteins involved in inflammation and immune regulation [40–45].

The inflammatory response of endothelial cells to pathogenic stimuli is a pivotal step in the development of AS. AS is a disease characterized by intra-arterial fat deposition and plaque formation. Several inflammatory factors, including MCP-1, vascular cell adhesion molecule-1 (VCAM-1), endothelial-leukocyte adhesion molecule (ELAM-1), and intercellular adhesion molecule-1 (ICAM-1), are involved in the initiation and progression of AS. MCP-1 recruits immune cells, such as monocytes, to the inflammatory site. VCAM-1 and ICAM-1 are adhesion molecules that enable immune cells to attach to the vascular wall and migrate into the subendothelial space, where they differentiate into macrophages. These activated macrophages internalize ox-LDL particles and subsequently differentiate into lipid-laden foam cells, a feature of early atherosclerotic lesions. Meanwhile, inflammatory macrophages secrete cytokines, such as interleukin-6, MCP-1, and TNF-α, which interact with other immune cells to promote plaque formation and exacerbate AS [46].

Mitogen-activated protein kinases (MAPKs) are fundamental signaling pathways involved in cellular responses across various cell types. Research indicates that rhein inhibits MCP-1 secretion via the p38 MAPK signaling pathway and suppresses the transcription and expression of VCAM-1 and ICAM-1, suggesting its potential as a preventive agent for AS [43,47]. Additionally, rhein suppresses the activation of Toll-like receptor (TLR), protein kinase B (Akt), and c-Jun N-terminal kinase MAPK signaling pathways, providing additional mechanisms for its anti-inflammatory action [44].

The nuclear factor-kappa B (NF-κB) signaling pathway, a cornerstone of inflammatory regulation [48], is intricately modulated by the TLR family, with TLR2 serving as a key activator of NF-κB-driven pro-inflammatory responses. Foundational studies have demonstrated that rhein exerts anti-inflammatory effects by inhibiting the TLR2/NF-κB axis [49,50]. However, a groundbreaking study revealed mechanistic complexity: while rhein at effective concentrations effectively suppresses NF-κB activation through targeting IκB kinase β (IKKβ), it simultaneously triggers IKKβ-mediated Caspase-1 activation, thereby enhancing secretion of the pro-inflammatory cytokines IL-1β and high mobility group box 1, a late-phase inflammatory mediator. This bidirectional regulatory pattern—exerting anti-inflammatory effects via NF-κB inhibition while promoting inflammatory cytokine maturation through Caspase-1 activation—highlights the dual role of IKKβ in inflammation: functioning as a pro-inflammatory regulator through the canonical NF-κB pathway while participating in posttranscriptional cytokine processing [45]. Furthermore, the dose-dependent effects of rhein introduce additional complexity. Low concentrations (< 10 μM) fail to inhibit NF-κB, whereas higher doses (> 20 μM) suppress NF-κB suppression [51]. This reflects a non-linear concentration threshold for pharmacological action: only when rhein reaches a sufficient concentration can it bind to target molecules (e.g., IKKβ) adequately to disrupt NF-κB activation—a classic ligand-receptor interaction feature requiring a minimum concentration to elicit biological responses.

Furthermore, studies have reported that in influenza A virus models, rhein antiviral efficacy exhibits significant host metabolic state dependency: Using MDCK cells and A549 lung cancer cells as hosts, researchers demonstrated that rhein alleviates viral replication-associated inflammation by inhibiting TLR4/Akt/p38/JNK pathways. However, its therapeutic effects are modulated by host redox status—exogenous oxidative stress (e.g., H_2_O_2_) reverses its protective benefits. Mechanistically, influenza A virus infection disrupts host redox homeostasis (elevated reactive oxygen species (ROS)/MDA levels and depleted GSH), while rhein partially restores this balance through antioxidant activity. Notably, under oxidative conditions, rhein inhibition of pro-inflammatory signaling (e.g., TLR4/NF-κB pathways) is attenuated. Consequently, these findings highlight that host metabolic state, particularly redox equilibrium, serves as a critical determinant for the antiviral outcomes of rhein, suggesting the need to integrate antioxidant strategies for optimized clinical application [44]. Beyond Caspase-1 and IKKβ, rhein may engage yet-unidentified regulators of inflammatory cascades, such as alternative kinases or transcription factors, which warrant further investigation.

In summary, as an inflammation regulator, the efficacy of rhein is influenced by multiple factors, including the cellular microenvironment, dosage, and disease stage. Its anti-inflammatory mechanism exhibits complex and diverse characteristics. It can exert anti-inflammatory effects by inhibiting specific inflammation-related signaling pathways, while it may also trigger pro-inflammatory responses due to changes in dosage and other factors. These findings facilitate a deeper understanding of the complexity of the inflammatory regulatory network, providing crucial evidence for the rational drug design targeting AS and fully highlighting the potential value of rhein in the treatment of relevant diseases.

#### Emodin

Emodin exerts its anti-AS effects through multiple mechanisms, primarily by inhibiting inflammation and regulating autophagy [52]. Animal experiments demonstrated that emodin significantly reduces serum inflammatory cytokine levels in mice [53,54]. Moreover, it can prevent early-stage inflammatory exudate, the increase in capillary permeability, and leukocyte migration, thereby exhibiting obvious anti-inflammatory effects [54]. Further mechanistic studies have revealed that emodin can inhibit the over-activation of key signaling molecules involved in immune responses and inflammation. These molecules are part of the "TLR/myeloid differentiation primary response protein 88/NF-κB" signaling axis [62]. The "TLR/myeloid differentiation primary response protein 88/NF-κB" signaling axis is a well-established signaling pathway that orchestrates pro-inflammatory gene expression and modulates immune-inflammatory responses. Emodin inhibits the inflammatory response within AS plaques by reducing the accumulation of lipids and macrophages and decreasing the expression of pro-inflammatory molecules. As a result, it enhances the stability of plaques and reduces the risk of plaque rupture and the trigger of further cardiovascular events [55].

In addition to its proven anti-inflammatory effects, the regulation of autophagy (a key cellular process with environment-dependent effects in AS) by emodin is the core mechanism by which it exerts its anti-atherosclerotic activity. In macrophages, emodin enhances autophagic flux by upregulating the expression of autophagy-related proteins LC3-II and Beclin-1, thereby accelerating the lysosomal clearance of oxidized low-density lipoprotein (ox-LDL) (a primary driver of foam cell formation) and dysfunctional mitochondria (a major source of ROS). This enhanced autophagic clearance reduces intracellular ROS accumulation, dampens pro-inflammatory cytokine release, and inhibits macrophage-to-foam cell transformation—all pivotal steps in halting early atherogenesis [56,57]. Similarly, in VSMCs, emodin-induced autophagy facilitates the elimination of misfolded proteins and damaged organelles, which is essential for preserving the contractile phenotype of VSMCs (rather than their pathological synthetic phenotype) and thus maintaining the structural stability of atherosclerotic plaques [58]. Complementing this autophagic regulation, macrophages’ phagocytic capacity—specifically efferocytosis (the clearance of apoptotic cells) and the uptake of modified lipoproteins—plays a fundamental role in sustaining vascular homeostasis: defective phagocytosis leads to the accumulation of necrotic debris and ox-LDL within plaques, triggering sustained pro-inflammatory responses and exacerbating foam cell formation, which are hallmarks of early AS pathogenesis [59]. Importantly, the impact of emodin-regulated autophagy is tightly linked to AS progression stages: in early lesions, tempered autophagic flux in macrophages and VSMCs supports the clearance of ox-LDL and dysfunctional mitochondria, curbing foam cell generation and preserving plaque stability [60]; in advanced plaques, however, emodin can have dual regulatory effects on autophagic initiation and flux. These effects may induce excessive autophagy, which, paradoxically, precipitates macrophage apoptosis and expands the necrotic core of the plaque. This emphasizes the necessity of stage-specific autophagy modulation to optimize therapeutic outcomes. Furthermore, under hyperglycemic conditions, emodin maintains endothelial barrier function by restoring disrupted autophagic balance, offering targeted protection for diabetic patients at high risk of AS [61]. Collectively, these findings emphasize that adequate, context-appropriate autophagy mitigates AS progression by sustaining cellular and vascular homeostasis, while dysregulated autophagy (either insufficient or excessive) drives lesion development.

The mammalian target of rapamycin (mTOR) pathway, a central regulator of autophagy, exhibits context-dependent dual roles in AS [63,64]. Under physiological conditions, mTOR suppresses autophagy by phosphorylating downstream effectors, while its dysregulation in AS creates a paradoxical scenario where either chronic inhibition or hyperactivation accelerates disease progression. Emodin, a natural compound, has been shown to induce autophagy by inhibiting the PI3K/Akt/mTOR axis [65]. Mechanistically, it reduces Akt phosphorylation via PI3K suppression, thereby inhibiting mTOR and activating autophagy-related proteins (e.g., LC3, Beclin-1). This process clears damaged cellular components and mitigates foam cell formation in macrophages, offering therapeutic potential [66].

Emodin exhibits a time- and dose-dependent regulation of the mTOR signaling pathway. In human hepatocyte L02 cells, the inhibitory effect of emodin on the PI3K/Akt/mTOR pathway intensifies over time. Notably, related protein phosphorylation declines at 2 h, drops significantly at 4 h, and shows remarkable changes at 6–8 h. Moreover, emodin dose-dependently suppresses the phosphorylation of the pathway in the 10–40 μM range [65]. In *ApoE-/-* mice with AS induced by a high-fat diet, medium and high doses of emodin (20–40 mg/(kg·d)) demonstrate time-dependent mTOR inhibition and AS improvement. Specifically, lesions improve slightly at 4 weeks, plaque area decreases significantly at 8 weeks, and the therapeutic effects peak at 12 weeks [67,68]. In high-glucose-treated podocytes, emodin significantly modulates the Adenosine 5'-monophosphate (AMP)-activated protein kinase (AMPK)/mTOR-mediated autophagy pathway after 6 h of treatment in a concentration-dependent manner [69]. Overall, these studies indicate that the regulation of the mTOR pathway by emodin is time-and dose-dependent, highlighting its potential as a therapeutic agent for diseases related to mTOR dysfunction.

#### Rhaponticin

Rhaponticin exhibits multifaceted anti-inflammatory effects that are pivotal in the pathogenesis of AS. It significantly reduces the levels of key pro-inflammatory cytokines, namely TNF-α, interleukin-1β, and interleukin-8. Meanwhile, it modulates the role of interleukin-10, an anti-inflammatory cytokine. Although interleukin-10 is generally recognized as an anti-inflammatory mediator, within the overall anti-inflammatory context relevant to AS, it likely plays a crucial part in fine-tuning the inflammatory milieu to maintain a balanced state that opposes the progression of AS. At the molecular level, within the context of AS-related processes, rhaponticin concurrently suppresses the mRNA expression of interleukin-1β and transforming growth factor-β (TGF-β). It also downregulates the protein levels of caspase-1 and cyclooxygenase-2. Moreover, it inhibits the activation of the NOD-like receptor family pyrin domain containing 3 (NLRP3) inflammasome. Collectively, these actions effectively attenuate the inflammation associated with AS [70]. The therapeutic potential of rhaponticin in AS is further substantiated by the study conducted by Li et al. [71]. In an AS-related model, they demonstrated that rhaponticin can inhibit the NF-κB and MAPK signaling pathways. The activation of these pathways is closely associated with the production of pro-inflammatory mediators in AS. By blocking both pathways, rhaponticin reduces the activity of inducible nitric oxide synthase (iNOS) and cyclooxygenase-2. This, in turn, effectively disrupts the pro-inflammatory cascade that drives the progression of atherosclerotic lesions, highlighting its potential as a therapeutic agent for AS.

#### Aloe-emodin

Emerging evidence indicates that aloe-emodin possesses significant anti-inflammatory properties relevant to AS. This anthraquinone derivative effectively suppresses the release of key inflammatory mediators including nitric oxide (NO), TNF-α, and interleukin-1β, while downregulating microRNA-33-a critical regulator of inflammatory responses [72,73]. The anti-inflammatory effects are particularly notable in the context of NO regulation. During inflammation, proinflammatory cytokines preferentially induce iNOS over other NOS isoforms (endothelial and neuronal NOS), leading to excessive NO production. Aloe-emodin counteracts this process by suppressing iNOS expression, thereby reducing inflammatory NO levels. Mechanistically, this may occur through inhibition of LPS-induced extracellular signal-regulated kinase (ERK) phosphorylation, a pivotal event in proinflammatory signaling transduction [72]. These findings collectively position aloe-emodin as a multi-target anti-inflammatory agent capable of modulating both cytokine networks and enzymatic pathways involved in AS pathogenesis.

#### Other components

The anti-inflammatory repertoire of rhubarb extends beyond the previously characterized compounds. Chrysophanol has been shown to effectively alleviate inflammation through suppression of TNF-α production, as demonstrated in both cellular and animal models [74]. Physcion, another pharmacologically active anthraquinone, exerts multi-targeted effects by interfering with Akt phosphorylation-a master regulator of cellular metabolic pathways. This primary inhibition leads to downstream modulation of ERK and mTOR signaling cascades, resulting in coordinated anti-inflammatory responses [75]. Gallic acid further enhances the anti-inflammatory effects of rhubarb through the combined effect of inhibiting MAPK/NF-κB activation and stimulating nuclear factor erythroid 2-related factor 2 (Nrf2)-mediated ROS clearance. This multifaceted action collectively reduces the secretion of inflammatory cytokines and chemokines, decreases adhesion molecule expression, and limits inflammatory cell infiltration, ultimately suppressing the inflammatory cascade [76]. The synergistic interplay among these diverse bioactive components underscores the unique therapeutic potential of rhubarb in targeting the complex inflammatory networks underlying AS progression.

### Anti-oxidative stress

In addition to the anti-inflammatory mechanisms of rhubarb and its compounds discussed earlier, oxidative stress due to overproduction of ROS has been identified as a critical contributor to the pathogenesis of AS. Inflammation and oxidative stress are intricately linked in the pathogenesis of AS, and the antioxidant properties of rhubarb and its active components may work in concert with their anti-inflammatory effects to slow down the progression of AS.

AS is now regarded as a chronic inflammatory condition. Oxidative stress, triggered by an overabundance of ROS, plays a crucial role in its pathogenesis [77]. ROS, a group of highly active molecules, trigger a series of AS-promoting events, such as inflammation, endothelial dysfunction, and disturbed lipid metabolism [78]. For example, ROS can activate redox-sensitive transcription factors like NF-κB. Once activated, NF-κB promotes the expression of pro-inflammatory cytokines and adhesion molecules, which then facilitate the recruitment of immune cells to the arterial wall and the formation of atherosclerotic plaques.

Rhubarb, along with its active constituents, possesses the ability to scavenge free radicals and demonstrate antioxidant effects, thereby counteracting the harmful impacts of various ROS [79]. These components may exert their antioxidant actions through multiple pathways. They can directly neutralize ROS, enhance the activity of endogenous antioxidant enzymes like catalase (CAT), superoxide dismutase (SOD), and glutathione peroxidase (GSH-Px), and regulate redox-sensitive signaling pathways to restore the cellular redox equilibrium.

**Fig. 4:**
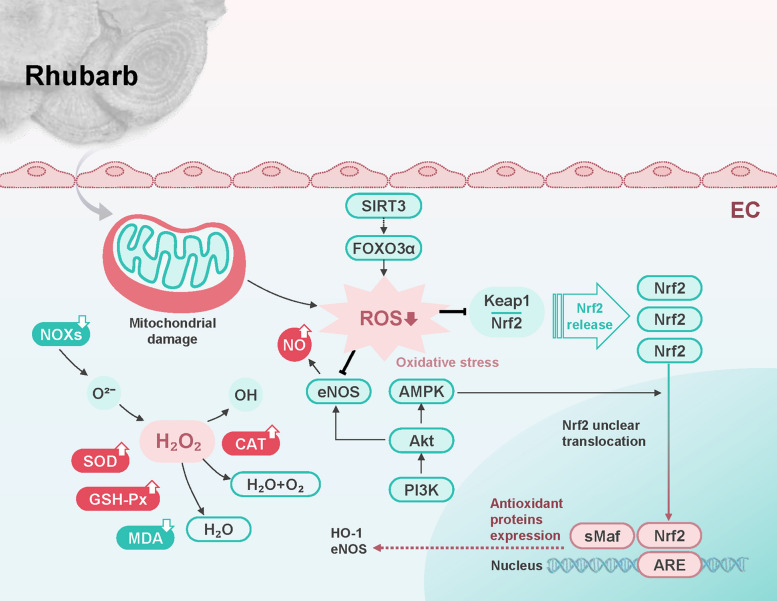
Antioxidant Pathways of Rhubarb. Rhubarb and its compounds exhibit significant antioxidant effects through multiple pathways. They strongly scavenge excess ROS and H_2_O_2_ from intracellular mitochondrial damage, while increasing serum CAT levels and SOD, GSH-Px activities, reducing MDA levels, and inhibiting NADPH oxidase production. Additionally, rhubarb activates the PI3K/Akt/AMPK pathway to promote Nrf2 release from Keap1 and nuclear translocation, enhancing antioxidant enzyme (HO-1, eNOS) expression. It also inhibits oxidative stress via activating the SIRT3/FOXO3α signaling pathway. Akt, protein kinase B; AMPK, Adenosine 5'-monophosphate (AMP)-activated protein kinase; ARE, antioxidant response element; CAT, catalase; EC, endothelial cell; eNOS, endothelial nitric oxide synthase; FOXO3α, forkhead box O3α; GSH-Px, glutathione peroxidase; HO-1, heme oxygenase-1; MDA, malondialdehyde; NADPH, nicotinamide adenine dinucleotide phosphate; NO, nitric oxide; NOXs, NADPH oxidases; Nrf2, nuclear factor erythroid 2-related factor 2; PI3K, phosphatidylinositol 3-kinase; ROS, reactive oxygen species; SIRT3, sirtuin 3; sMaf, small Maf protein; SOD, superoxide dismutase

These discoveries indicate that the antioxidant effects of rhubarb and its active components show great potential in the treatment of AS. However, further research is necessary to clarify the precise molecular mechanisms underlying their antioxidant actions, to assess their clinical efficacy in large-scale human studies, and to investigate their possible synergistic effects with currently available anti-atherosclerotic therapies (Fig. 4).

#### Emodin and physcion

Emodin confers protection against oxidative stress in AS through coordinated metabolic and vascular modulation. It significantly reduces established AS risk factors including triglycerides (TG) and total cholesterol (TC), while concurrently lowering serum aspartate aminotransferase and alanine aminotransferase levels-biomarkers reflecting hepatic protection during AS prevention. At the molecular level, emodin downregulates cytochrome P450 2E1 expression, a pivotal enzyme in oxidative stress generation, thereby decreasing ROS production. This is complemented by reduced thiobarbituric acid reactive substances, indicating attenuated lipid peroxidation damage. The vascular protective effects of emodin are evidenced by its inhibition of α-smooth-muscle actin and collagen type I expression, counteracting arterial wall thickening and fibrosis characteristic of advanced AS [80,81]. Comparative quantum chemical analyses show that emodin has a slightly stronger antioxidant capacity than physcion. This difference is attributed to the distinct electronic properties of the phenolic hydroxyl groups in their molecular structures [82]. Physcion, however, exhibits exceptional radical scavenging ability against 2,2-diphenyl-1-picrylhydrazyl, surpassing the efficacy of vitamin C. Notably, it shows context-dependent pro-oxidant effects in neutrophils under photostimulation [83,84]. Mitochondrial studies further substantiate the antioxidant profile of emodin. Emodin protects SH-SY5Y human neuroblastoma cells (a commonly used neuronal model) from methylglyoxal-induced damage. It achieves this by restoring ATP levels and membrane potential, while simultaneously blocking ROS accumulation [85]. Additionally, emodin modulates oxidative stress-responsive pathways including peroxisome proliferator-activated receptor gamma (PPARγ), and the Nrf2/heme oxygenase-1 axis, demonstrating context-specific regulation of cellular defense mechanisms against AS-associated oxidative stress and inflammation [80,86].

#### Rhein

In addition to emodin and physcion, another important component of rhubarb with antioxidant properties is rhein. Rhein demonstrates potent antioxidant activity through multifaceted enzymatic regulation and cellular protection. It significantly enhances endogenous antioxidant defenses by elevating SOD and GSH-Px activity while reducing malondialdehyde levels, a key marker of lipid peroxidation [87]. Beyond direct free radical scavenging, rhein mitigates oxidative stress in tissue cells through three interconnected mechanisms: inhibition of NADPH oxidase activity (the primary source of cellular ROS), reduction of intracellular peroxide accumulation, and protection of pancreatic β-cell function and renal tubular epithelial cells against oxidative damage [88]. Notably, the antioxidant effects of rhein intersect with its anti-fibrotic properties. Wu et al. [89]. demonstrated that rhein inhibits TGF-β1-induced epithelial-mesenchymal transition—a pivotal process in tissue fibrosis—through activation of the sirtuin 3 (SIRT3)/forkhead box O3α (FOXO3α) pathway. This pathway serves as a central controller of cellular redox balance and longevity, suggesting the potential of rhein to modulate fundamental aging-related processes. By concurrently suppressing oxidative stress and activating SIRT3/FOXO3α signaling, rhein establishes a dual protective mechanism against both metabolic and fibrotic complications in chronic inflammatory diseases like AS [89].

#### Chrysophanol

Chrysophanol demonstrates significant antioxidant activity by enhancing cellular defense systems. Studies have confirmed its ability to increase the activity of antioxidant enzymes, including SOD, GSH-Px, and CAT, which are essential for neutralizing free radicals and preventing oxidative damage [90]. The mechanism involves activation of the Nrf2 signaling pathway, as demonstrated by Lian et al. [91] in LPS-treated cells, where chrysophanol effectively reduced ROS production. Notably, chrysophanol has shown neuroprotective potential in models of amyloid β-induced toxicity. It reduces elevated levels of malondialdehyde, a marker of oxidative stress and lipid peroxidation, while restoring the activity of SOD, GSH-Px, and CAT in the brain. These effects are associated with improved cognitive function, particularly spatial memory, in experimental animals [92]. The findings suggest chrysophanol may help counteract oxidative damage in neurodegenerative conditions.

#### Other components

The PI3K and its downstream effector Akt form a pivotal signaling cascade that governs a myriad of biological processes, including cell growth, proliferation, apoptosis, and protein synthesis [93]. The PI3K/Akt signaling cascade has been shown to participate in Nrf2-dependent antioxidant defense mechanisms [94]. AMPK, a heterotrimeric serine/threonine kinase, serves as a crucial energy sensor within cellular metabolism. It responds to a diverse array of metabolic stresses, such as oxidative stress, inflammation, and hypoxia [95]. By stimulating the nuclear translocation of Nrf2 and suppressing inflammation through the inhibition of the NF-κB signaling pathway, AMPK plays a vital role in cellular stress management [96]. Studies have demonstrated that gallic acid significantly attenuates the LPS-induced expression of inflammatory mediators and reduces ROS levels. It achieves these effects by activating the Akt/AMPK/Nrf2 signaling axis, thereby enhancing the expression of antioxidant-related genes [97]. Moreover, gel dressings containing gallic acid as the primary active component promote wound healing. They do so by upregulating the expression of antioxidant genes and growth factors, while concurrently downregulating the expression of pro-inflammatory genes [98,99]. Rhaponticin also exhibits a potent antioxidant capacity, effectively scavenging intracellular ROS, 1,1-diphenyl-2-picrylhydrazyl radicals, and hydrogen peroxide [100,101]. All these active components of rhubarb are intimately associated with oxidative stress and cellular damage. These findings collectively suggest that rhubarb has antioxidant properties.

### Regulation of lipid metabolism

**Fig. 5:**
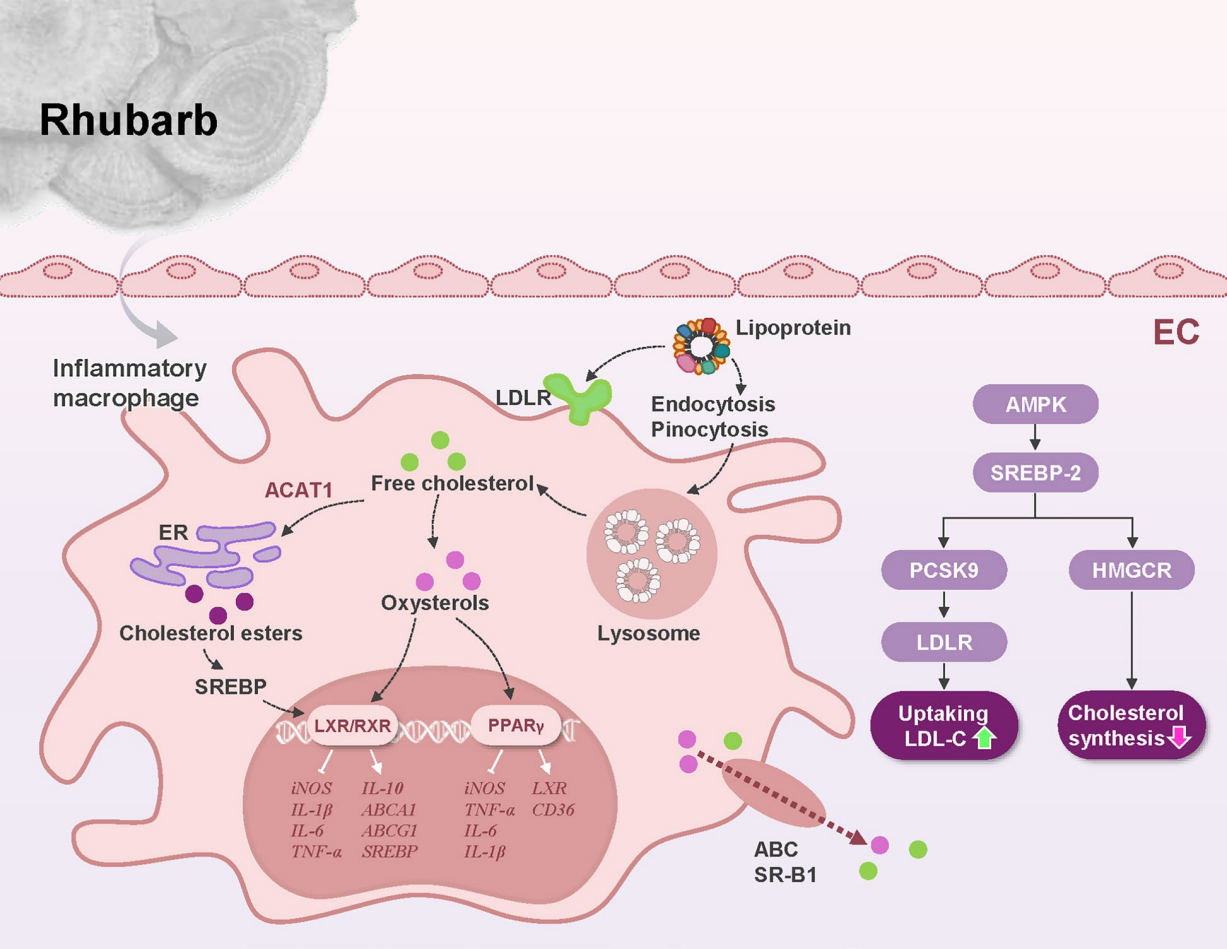
Lipid metabolism pathways of rhubarb. Rhubarb and its compounds modulate lipid metabolism via signaling pathways including PPARγ and AMPK/SREBP-2. Lipoproteins are internalized through pinocytosis or LDLR-mediated endocytosis, then delivered to lysosomes for degradation. Within cells, endocytosed lipoproteins and cholesterol undergo sequential metabolic processes: ACAT1 esterifies free cholesterol to cholesteryl esters in the endoplasmic reticulum, preventing free cholesterol accumulation. SREBP serves as a key regulator in cholesterol metabolism, while SR-B1 and ABC facilitate cholesterol efflux to maintain intracellular cholesterol homeostasis. Additionally, LXR/RXR supports lipid metabolism regulation by specifically activating transcription of cholesterol efflux-related genes, effectively reducing excessive intracellular cholesterol accumulation. ABC, ATP-binding cassette; ABCA1, ATP-binding cassette transporter A1; ABCG1, ATP-binding cassette transporter G1; ACAT1, acyl-CoA:cholesterol acyltransferase 1; AMPK, Adenosine 5'-monophosphate (AMP)-activated protein kinase; CD36, cluster of differentiation 36; EC, endothelial cell; ER, endoplasmic reticulum; HMGCR, 3-hydroxy-3-methylglutaryl-CoA reductase; iNOS, inducible nitric oxide synthase; IL-1β, interleukin-1β; IL-6, interleukin-6; IL-10, interleukin-10; LDL-C, LDL-cholesterol; LDLR, low-density lipoprotein receptor; LXR, liver X receptor; PCSK9, proprotein convertase subtilisin/kexin type 9; PPARγ, peroxisome proliferator-activated receptor gamma; RXR, retinoid X receptor; SREBP, sterol regulatory element-binding protein; SR-B1, scavenger receptor class B type 1; TNF-α, tumor necrosis factor-alpha

AS is characterized by arterial wall thickening and hardening due to the accumulation of lipids, cholesterol, and inflammatory components [102]. Modern lifestyle changes, particularly dietary shifts and reduced physical activity, have contributed to rising global hyperlipidemia prevalence. Chronic dyslipidemia disrupts metabolic homeostasis, promoting atherosclerotic plaque formation that impairs blood flow and elevates cardiovascular risk. Growing evidence highlights the unique potential of rhubarb in lipid metabolism modulation and AS prevention [103]. Its multi-component system targets diverse pathological processes, offering novel therapeutic strategies grounded in traditional medicine wisdom and modern pharmacological research. The following sections will systematically examine these mechanisms (Fig. 5).

#### Emodin

Emodin has emerged as a promising therapeutic agent for modulating lipid metabolism and preventing AS. Comprehensive studies have demonstrated that emodin can significantly improve serum lipid profiles by reducing TG, TC, and LDL levels, while simultaneously elevating high-density lipoprotein concentrations and SOD activity. Notably, emodin enhances reverse cholesterol transport through promoting cholesterol efflux from peripheral tissues, thereby contributing to atherosclerotic plaque regression [104–108,115].

The liver, being the central regulator of lipid metabolism, serves as a primary target for the therapeutic effects of emodin [109]. Molecular investigations reveal that emodin orchestrates a complex regulatory network, which involves two key mechanisms: first, the upregulation of AMPKα and low-density lipoprotein receptor (LDLR) expression; second, the enhancement of ATP-binding cassette transporter A1 and ATP-binding cassette transporter G1 activity. Emodin suppresses key regulators of cholesterol biosynthesis. These include sterol regulatory element-binding protein 2, proprotein convertase subtilisin/kexin type 9 (PCSK9), and 3-hydroxy-3-methylglutaryl-CoA reductase. This multifaceted modulation collectively improves hepatic lipid handling and reduces atherosclerotic risk indices [107,110,111].

Of particular significance is the activation of PPARγ by emodin, a nuclear receptor highly expressed in lipid-laden foam cells [116]. As a transcription factor activated by ligand binding, PPARγ regulates genes involved in lipid metabolism and inflammation. Emodin-mediated PPARγ activation upregulates ATP-binding cassette transporter A1 expression—a critical mediator of cholesterol efflux from foam cells to apolipoprotein A-I. This process facilitates reverse cholesterol transport, reducing intracellular cholesterol accumulation and promoting plaque stability [110].

Beyond lipid metabolism, glycolipid metabolism is crucial for energy production and material sources [112]. Emodin exhibits beneficial effects on glucose homeostasis by enhancing adipocyte glucose utilization and improving insulin sensitivity, mechanisms that indirectly contribute to AS prevention [113,114]. These metabolic improvements are mediated through various pathways including endothelial nitric oxide synthase (eNOS)/NO system activation and AMPK/sterol-regulatory element-binding protein 2 signaling modulation [107,108,113,114].

#### Chrysophanol

Chrysophanol has been demonstrated to regulate lipid metabolism through multiple mechanisms. Research indicates that this anthraquinone derivative reduces intracellular TC and TG accumulation in adipocytes, while downregulating the expression of key adipogenic transcription factors such as PPARγ and CCAAT/enhancer-binding protein α. These molecular modifications are associated with decreased lipid droplet formation and improved systemic lipid balance [117,118]. The metabolic benefits of chrysophanol extend across different adipose depots. In studies using rhubarb-derived compounds, chrysophanol was found to activate thermogenic pathways in brown adipose tissue, enhancing energy expenditure and insulin sensitivity. Parallel investigations in white adipose tissue of high-fat diet models revealed that chrysophanol upregulates lipolytic genes while suppressing adipogenic genes, potentially through activation of the AMPK/sirtuin 6 signaling axis. These findings collectively suggest that chrysophanol may represent a promising therapeutic agent for metabolic disorders associated with obesity [119].

#### Rhein

Rhein, a key bioactive compound in rhubarb, demonstrates significant efficacy in regulating lipid metabolism. Experimental evidence [120] confirms its ability to reduce TC, LDL-C, and fasting blood glucose levels in high-fat diet models. The primary mechanism involves PPARγ antagonism, wherein rhein suppresses transcriptional activity and downregulates downstream target genes. PPARγ, a nuclear receptor governing lipid storage and adipocyte differentiation, exhibits context-dependent effects: physiological activation promotes lipid accumulation, whereas aberrant overactivation upregulates macrophage lipid uptake genes, accelerating foam cell formation—a critical event in AS plaque development [123]. By antagonizing PPARγ, rhein may mitigate lipid deposition in macrophages, thereby impeding foam cell generation and AS progression. Beyond PPARγ modulation, rhein targets liver X receptors, master regulators of cholesterol homeostasis. Liver X receptors activate cholesterol efflux genes (e.g., ATP-binding cassette transporter A1), reducing intracellular cholesterol overload and conferring cardiovascular protection [121]. Notably, rhein also upregulates uncoupling protein 1 in brown adipose tissue, offering dual benefits against obesity and metabolic disorders [122].

#### Aloe-emodin

Aloe-emodin shares structural homology with emodin, potentially conferring comparable bioactivities. Substantial evidence supports its efficacy in ameliorating hyperlipidemia and attenuating cardiovascular risks through multifaceted mechanisms. A seminal investigation [123] demonstrated that aloe-emodin treatment significantly decreased serum TC and LDL levels in hyperlipidemic murine models. This effect was principally mediated through modulation of the PCSK9/LDLR pathway, which serves as a master regulator of cholesterol homeostasis. The underlying mechanism involves PCSK9-mediated degradation of hepatic LDLR, thereby impairing LDL-cholesterol (LDL-C) clearance. Aloe-emodin potently inhibits PCSK9 expression, consequently stabilizing LDLR and augmenting hepatic LDL-C uptake, which culminates in marked hypolipidemic effects. Moreover, this anthraquinone derivative exhibits cardioprotective properties by suppressing high-fat diet-induced cardiac inflammation through selective inhibition of the TLR4/NF-κB signaling cascade [124], highlighting its dual therapeutic potential as both a lipid-lowering and anti-inflammatory agent.

#### Rhubarb polysaccharide

Emerging evidence indicates that rhubarb polysaccharides exhibit significant lipid-modulating properties. Studies have demonstrated their ability to simultaneously reduce serum levels of TG, TC, and LDL-C, while increasing high-density lipoprotein-C concentrations and enhancing SOD activity [125]. These combined effects contribute to improved lipid profiles and enhanced antioxidant capacity, suggesting a potential therapeutic role in managing dyslipidemia. The polysaccharides appear to exert these beneficial effects through multiple pathways, including modulation of hepatic lipid metabolism and reduction of oxidative stress, although the precise molecular mechanisms require further elucidation.

### Vascular protection

AS is a chronic inflammatory vascular disease characterized by endothelial dysfunction. This condition predominantly occurs in the subendothelial space of medium-sized arteries at sites with disturbed blood flow. It is triggered by the interplay between endothelial dysfunction and the retention of subendothelial lipoproteins [126,127]. In recent years, studies have demonstrated that rhubarb can enhance endothelial function in AS patients. It achieves this by decreasing plasma cholesterol levels and mitigating damage to vascular endothelial cells [128]. Moreover, the development of AS is closely associated with the phenotypic switching of VSMCs [129]. Rhubarb and its active components can inhibit VSMC phenotypic switching through multiple mechanisms. This action maintains arterial wall stability and reduces plaque formation/rupture risks, positioning them as promising AS therapeutics.

#### Aloe-emodin

Mounting evidence underscores the multifaceted therapeutic potential of aloe-emodin in AS. Tang et al. [130] demonstrated that aloe-emodin derivatives enhanced endothelial autophagy via beclin1-dependent pathways, suggesting its promise as an AS treatment candidate. Beyond promoting endothelial self-repair, Zhang et al. [131] uncovered a novel anti-inflammatory mechanism using a mouse AS model: aloe-emodin suppresses NLRP3 inflammasome activation by facilitating NLRP3 ubiquitination. This inhibition reduces the release of pro-inflammatory mediators, including IL-1β, IL-18, and the damage-associated molecular pattern protein high mobility group box 1, thereby preserving endothelial barrier integrity through restored tight junction protein expression (e.g., zonula occludens-1, occludin) and attenuated vascular permeability. Mechanistically, NLRP3 inflammasome activation—a key innate immune response driver in AS—triggers VSMC phenotypic switching from a contractile to synthetic state under pathological conditions [132]. Synthetic VSMCs exhibit enhanced proliferation, migration, and secretion of ECM proteins (e.g., collagen I/III) and inflammatory factors (e.g., MCP-1, TNF-α), which collectively promote fibrous cap thinning, ECM remodeling, and plaque instability [133]. By blocking NLRP3 activation, aloe-emodin disrupts this vicious cycle, reducing VSMC inflammatory factor expression and ECM deposition while improving endothelial function. These findings position aloe-emodin as a dual-acting agent capable of simultaneously inhibiting vascular inflammation and enhancing endothelial protection, offering a novel therapeutic strategy for cardiovascular diseases.

Furthermore, considering the significant impact of vascular calcification on the progression of AS and the prognosis of cardiovascular disease patients, aloe-emodin has also shown potential in this aspect. Significantly, vascular calcification greatly increases the morbidity and mortality risk in patients with cardiovascular diseases [134]. Studies have shown [135] that aloe-emodin not only suppresses the phenotypes of Ca^2+^-induced calcification but also reduces the level of calcium in VSMCs. Aloe-emodin attenuates calcification of VSMCs through the bone morphogenetic protein 2 (BMP2)-dependent mothers against decapentaplegic homolog 4 (SMAD4) signaling pathway. BMP2 is a key osteogenic protein in vascular calcification. SMAD4, as a crucial downstream signaling mediator of BMP2, participates in the regulation of various cellular processes, including the phenotypic transformation of VSMCs and the progression of vascular calcification. By modulating this signaling axis, aloe-emodin inhibits the osteogenic transdifferentiation of VSMCs, reduces pathological calcium deposition, and prevents vascular calcification, thereby providing a novel phytochemical approach for the intervention of AS.

#### Emodin

Emodin functions as a selective proliferation inhibitor of VSMCs, demonstrating inhibitory effects on TNF-α-induced proliferation of human aortic smooth muscle cells. The compound provides significant protection against oxidative damage in human umbilical vein endothelial cells and suppresses arterial intima-media formation, suggesting its potential as a therapeutic agent for preventing restenosis and intimal hyperplasia [136–138]. The vasoprotective effects of emodin involve modulation of the miR-126-mediated signaling pathway, which critically regulates vascular repair and endothelial homeostasis. In balloon-injured rat arteries, emodin attenuates intimal thickening through regulation of the Wnt4/Dvl-1/β-catenin cascade. Within this pathway, Wnt4 activates Dishevelled-1 protein, resulting in β-catenin stabilization and nuclear translocation. This process promotes transcription of genes associated with endothelial cell proliferation and survival, ultimately enhancing carotid intimal healing and reducing neointima formation. These findings underscore the therapeutic potential of emodin in vascular injury repair [139].

#### Rhein

Rhein demonstrates multifaceted protection on vascular endothelium through coordinated modulation of oxidative and fibrotic pathways. Experimental data show it significantly elevates NO and nitric oxide synthase (NOS) levels while enhancing SOD activity, collectively ameliorating H_2_O_2_-induced endothelial damage [142]. Mechanistically, rhein exerts rapid suppression on TGF-β1-induced plasminogen activator inhibitor-1 expression via inhibiting the TGF-β1/ERK1/2 MAPK cascade. As the p38 MAPK isoforms are central to cellular proliferation and stress responses, their overactivation by TGF-β1 drives pathological plasminogen activator inhibitor-1 overexpression and endothelial dysfunction. Rhein effectively attenuates ERK1/2 phosphorylation, thereby normalizing plasminogen activator inhibitor-1 levels and preserving vascular homeostasis [143].

#### Gallic acid

By modulating key metabolic pathways, gallic acid has emerged as a promising anti-AS compound. Experimental studies have shown that gallic acid is able to slow down cell cycle progression by activating AMPK, which subsequently phosphorylates and activates eNOS. This AMPK-mediated eNOS activation enhances NO production through the conversion of L-arginine to L-citrulline. The increased NO bioavailability exerts multiple anti-atherogenic effects, including vasodilation, suppression of VSMC proliferation, and inhibition of inflammatory adhesion molecule expression (e.g., VCAM-1/ICAM-1). These actions contribute to the stabilization of atherosclerotic plaques [144]. Moreover, NO counteracts oxidative stress by scavenging superoxide anions, further protecting plaque integrity [140[. Additionally, NO modulates immune responses within the vascular wall by suppressing macrophage activation and foam cell formation, key drivers of atherosclerotic progression. Its ability to mitigate endoplasmic reticulum stress and mitochondrial dysfunction further preserves endothelial homeostasis [141]. These pleiotropic actions underscore the central role of NO in maintaining vascular health and highlight the potential of gallic acid as a multifaceted therapeutic agent. The dual modulation of AMPK signaling and NO biosynthesis makes gallic acid a unique candidate for intervention in AS.

### Antithrombotic and hyperfibrinolytic effects

Thrombus formation occurs at sites of vascular injury through blood coagulation and platelet adhesion and aggregation, and it represents a critical pathological feature in ASCVD that correlates with increased thrombotic risk [145]. Recent work combining human plasma coagulation assays, fibrinolytic protein measurements, and endothelial cell hemostatic profiling revealed that rhubarb extract broadly alters the hemostatic behavior of plasma proteins and endothelial cells, conferring systemic anticoagulant activity [146]. The extract downregulates the expression of tissue-type plasminogen activator and plasminogen mRNAs, indicating dual regulatory effects on coagulation and fibrinolysis systems. This pharmacological profile aligns with TCM principles of promoting blood circulation without inducing hemorrhage while stopping bleeding without causing stasis. Furthermore, rhaponticin isolated from rhubarb has been identified as a prodrug exhibiting broad-spectrum antithrombotic activity along with antiallergic properties [147]. These findings suggest rhubarb-derived compounds hold promise for thrombotic disorder treatment.

## Efficacy and safety

Rhubarb, a cornerstone of TCM increasingly studied in modern contexts, presents a paradox in atherosclerotic disease management: its therapeutic potential is substantial, yet its safety risks demand rigorous scrutiny. This section synthesizes evidence on the mechanisms, toxicities, and risk-mitigation strategies of rhubarb, emphasizing the dialectical balance between efficacy and harm in clinical practice.

Growing data support the utility of rhubarb in AS. In dyslipidemic patients or those with unstable plaques, rhubarb micro-powders have shown lipid-lowering effects (15–20% reduction in LDL-C, 10–15% in TG over 6 months) comparable to statins in select cohorts, alongside imaging evidence of stabilized carotid plaques (reduced intima-media thickness, enhanced fibrous cap integrity) [152]. Mechanistically, wine-processed rhubarb modulates the iNOS/NO pathway to boost NO bioavailability and suppress pro-inflammatory cytokines (TNF-α, IL-6) [153]. TCM formulations like Modified Dahuang Mudanpi Decoction and Dahuang Mochong Pill further validate its synergistic value in multi-herb combinations, with the former reducing vulnerable plaques in carotid AS [161] and the latter achieving high efficacy rates in cerebral arteriosclerosis. These findings position rhubarb as a promising adjunct, particularly for statin-resistant patients or those requiring concurrent anti-inflammatory management.

However, the toxicity profile of rhubarb complicates its use, reflecting the broader challenge of potential toxicity in traditional botanicals [148,151]. Dose-dependent organ toxicity is observed in kidneys, livers, and reproductive systems: high-dose administration (16 g/kg in rats, tenfold clinical levels) induces renal impairment, while low doses (2 g/kg) remain safe [150,154]. Hepatotoxicity involves disrupted cAMP signaling, cholinergic dysfunction, and perturbed transient receptor potential channels [155,156], with prolonged use increasing drug-induced liver injury risk. Reproductive toxicity includes uterine/ovarian damage in juvenile female rats [158] and embryonic loss via uterine microenvironment disruption [159]. Age modulates susceptibility—geriatric rodents exhibit heightened renal sensitivity, while immature animals show relative resilience [160]—findings with implications for elderly patients. Additional concerns include gastrointestinal intolerance (15–30% at standard doses), drug-drug interactions (e.g., warfarin, increasing International Normalized Ratio by 1.5–twofold), and metabolic variability (e.g., CYP2C19 polymorphisms elevating emodin exposure by 40%), hindering dose optimization.

Navigating this balance requires patient-centered strategies aligned with TCM pharmacovigilance principles [149]: strict dose titration (3–6 g/day), vigilant monitoring of high-risk groups (elderly, pregnant individuals, those with renal/hepatic impairment), and novel delivery systems (e.g., pH-sensitive nanoparticles) to enhance plaque-specific accumulation while reducing systemic exposure. Standardization of processing (wine vs. raw) and extraction methods is critical to address variability [157], with validated protocols ensuring consistent marker levels (e.g., emodin > 5%) [153]. Long-term safety data (> 2 years) and pharmacogenomic/microbiome-guided personalized dosing are urgent priorities.

Clinically, the potential of rhubarb lies in its ability to complement conventional therapy, offering a multi-targeted approach (lipid modulation, inflammation suppression, plaque stabilization) that may benefit patients with complex atherosclerotic phenotypes. Translational impact hinges on overcoming current barriers: standardized formulations would enable reproducible clinical trials, while toxicity biomarkers and targeted delivery systems could mitigate risks. If these challenges are addressed, rhubarb may transition from a niche TCM remedy to an evidence-based adjunct, aligning traditional wisdom with modern precision medicine. Future research should focus on mechanistic translation from preclinical models, randomized trials of standardized products, and the development of toxicity early-warning systems to fully realize its therapeutic promise.

## Application prospects

Rhubarb, a cornerstone of TCM with growing global recognition, is rapidly advancing as a multifaceted therapeutic agent for AS management through interdisciplinary innovation. While preclinical studies highlight its potential via nanotechnology-enhanced drug delivery, gut microbiota modulation, and multi-omics pathway elucidation, clinical translation remains constrained by interconnected experimental and methodological barriers. Fundamental research often relies on oversimplified models: monocellular cultures fail to capture the multicellular dynamics of plaque microenvironments, while *ApoE-/-* murine systems inadequately replicate human coronary pathology, particularly complications like plaque rupture. Mechanistic investigations frequently suffer from incomplete pathway analysis, with critical regulators such as mTOR signaling or VSMC phenotypic switching remaining understudied, thereby limiting molecular-level insights. Clinical evidence is similarly fragmented, dominated by small-scale observational studies lacking standardized formulations, validated outcome measures, or long-term safety data essential for addressing hepatotoxicity and drug-drug interaction risks. Nanodelivery systems face technological hurdles, including poor reproducibility of synthesis protocols, suboptimal release kinetics, and inconsistent methods for assessing plaque penetration—all of which complicate regulatory approval and clinical translation.

Bridging these gaps demands targeted, interdisciplinary strategies. In basic science, the adoption of 3D-bioprinted atherosclerotic organoids and advanced imaging techniques—such as two-photon microscopy with fluorescently labeled metabolites—could clarify cell-type-specific mechanisms and plaque-microenvironment interactions. For nanodelivery, collaborations between materials scientists and pharmaceutical engineers are essential to develop dual-targeted, stimuli-responsive carriers (e.g., pH-sensitive nanoparticles with cleavable PEGylated coatings), which are optimized via machine learning algorithms to ensure consistent fabrication and controlled drug release. Clinically, large-scale, multi-center randomized controlled trials using supercritical CO_2_-extracted rhubarb formulations (standardized to > 50% emodin content) are imperative, incorporating diverse patient cohorts, combined hard endpoints (e.g., major adverse cardiovascular events) and surrogate markers (e.g., carotid intima-media thickness), alongside long-term safety monitoring (≥ 5 years). Regulatory harmonization through international consortia could establish quality control standards for plant-derived nanomaterials, addressing fragmentation in safety protocols. Finally, integrating multi-omics data (genomics, proteomics, metagenomics) with real-world evidence registries would enable precision medicine approaches, validating the therapeutic potential of rhubarb through systematic basic-translational-clinical integration.

In conclusion, the evolution of rhubarb from a TCM staple to a globally recognized cardiovascular therapy hinges on overcoming current methodological and translational barriers. By merging traditional pharmacognosy with cutting-edge technologies, such as CRISPR-based target validation, AI-driven nanocarrier design, and microbiome engineering, researchers in the field can unlock the multi-targeted anti-atherosclerotic potential of rhubarb. However, this transformation requires sustained investment in rigorous experimental design, cross-disciplinary collaboration, and global regulatory alignment, ensuring that historical wisdom converges with modern innovation to deliver sustainable, evidence-based solutions for AS management.

## Conclusion

This review underscores the unique therapeutic potential of natural bioactive compounds derived from rhubarb in managing AS, highlighting their diverse pharmacological properties and multifaceted mechanisms of action. Accumulating evidence demonstrates that rhubarb and its principal constituents—including anthraquinones (emodin, rhein, aloe-emodin, physcion, chrysophanol, etc.), tannins, and polysaccharides—exert significant anti-atherosclerotic effects. These phytochemicals operate through intricate, multi-target pathways, with anti-inflammatory and antioxidant activities forming the core of their protective mechanisms. Beyond these primary actions, they contribute to comprehensive cardiovascular regulation by modulating lipid metabolism, preserving vascular endothelial integrity, and inhibiting thrombotic processes. Both preclinical and clinical studies validate their capacity to intervene directly in AS pathogenesis while promoting systemic cardiovascular health. Given their polypharmacological profile, these natural products present promising prospects for developing innovative AS therapies, either as standalone interventions or synergistic adjuncts to conventional pharmacotherapies, offering enhanced efficacy and reduced adverse effects in cardiovascular disease management.

## Data Availability

No datasets were generated or analysed during the current study.

## Abbreviations

Akt: Protein kinase B
AMPK: Adenosine 5′-monophosphate (AMP)-activated protein kinase
AS: Atherosclerosis
ASCVD: Atherosclerotic cardiovascular disease
BMP2: Bone morphogenetic protein 2
CAT: Catalase
eNOS: Endothelial nitric oxide synthase
ERK: Extracellular signal-regulated kinase
GSH-Px: Glutathione peroxidase
ICAM-1: Intercellular adhesion molecule-1
IKKβ: IκB kinase β
iNOS: Inducible nitric oxide synthase
LDL: Low-density lipoprotein
LDL-C: LDL-cholesterol
LDLR: Low-density lipoprotein receptor
LPS: Lipopolysaccharide
MAPK: Mitogen-activated protein kinase
MCP-1: Monocyte chemoattractant protein-1
mTOR: Mammalian target of rapamycin
NF-κB: Nuclear factor-kappa B
NLRP3: NOD-like receptor family pyrin domain containing 3
Nrf2: Nuclear factor erythroid 2-related factor 2
NO: Nitric oxide
NOS: Nitric oxide synthase
ox-LDL: Oxidized low-density lipoprotein
PCSK9: Proprotein convertase subtilisin/kexin type 9
PI3K: Phosphatidylinositol 3-kinase
PPARγ: Peroxisome proliferator-activated receptor gamma
ROS: Reactive oxygen species
SOD: Superoxide dismutase
TC: Total cholesterol
TCM: Traditional Chinese medicine
TG: Triglycerides
TGF-β: Transforming growth factor-β
TLR: Toll-like receptor
TNF-α: Tumor necrosis factor-α
VSMC: Vascular smooth muscle cell
VCAM-1: Vascular cell adhesion molecule-1
